# Supplementary material for: The Effect of Cultivation Passaging on the Relative Telomere Length and Proliferation Capacity of Dental Pulp Stem Cells
Source: Biomolecules. 2021 Mar 20;11(3):464. doi: 10.3390/biom11030464 (PMC8035981; doi:10.3390/biom11030464)
Supplement: Supplementary file 1 [file biomolecules-11-00464-s001.pdf]

Supplementary Materials:

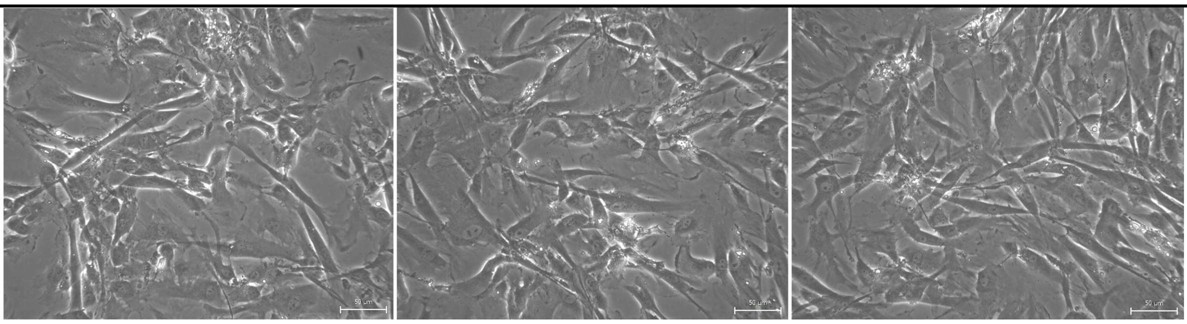

(**a1**) (a2) (a3)

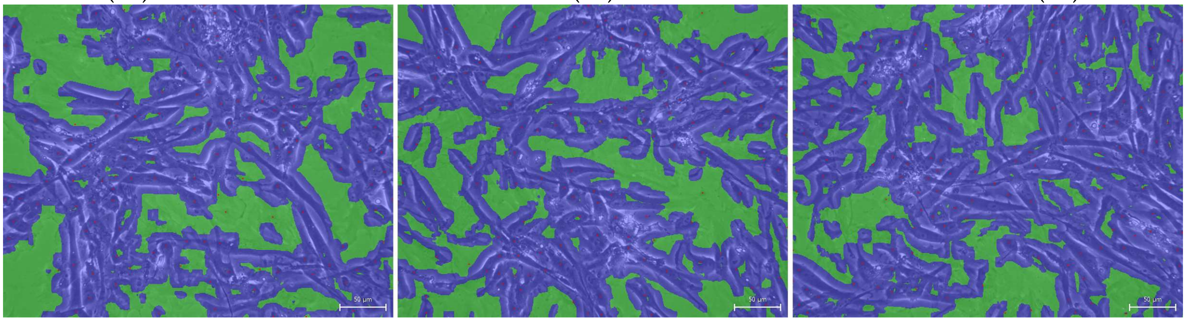

(b**1**) (b2) (b3)

(c) Table S1: Result table of the cell confluence measurement using the CKX-CCSW Confluency Checker (Olympus, Tokyo, Japan).

No.	Lineage	Count (Cells)	Confluency (%)
1	Z03_1	143	66.2%
2	Z03_2	140	66.8%
3	Z03_3	147	73.9%
Average:		143	68.9%
Estimation:		724 000	68.9%

(c)

Figure S1: The measurement of the 70 % cell confluence using the CKX-CCSW confluency checker software (Olympus, Tokyo, Japan). Lineage Z03B. Scale bar 50 µm. (a 1-3) Adherent DPSCs in an optic phase microscope before their passaging. (b 1-3) Confluency analysis (cells are marked as blue areas, cell to cell background as green areas) (c): The result table,

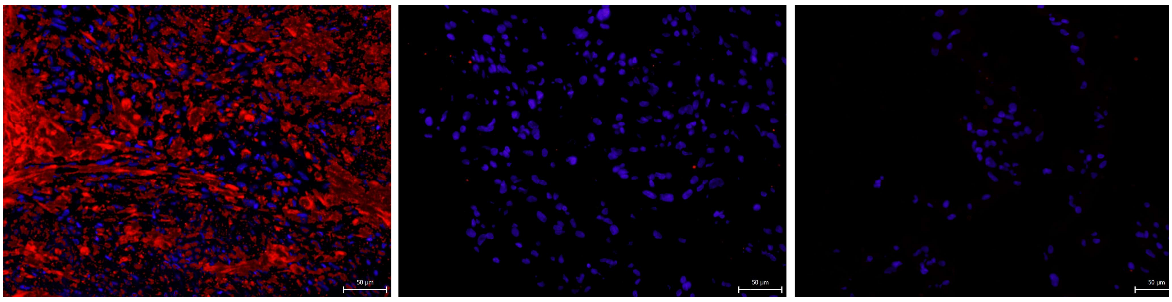

(**a**) (b) (c)

Figure S2: The type II collagen (red areas) in the extracellular mass and cell nuclei (blue areas). Scale bar 50 µm. (a) Analyzed in the cultivated DPSCs in the chondrogenic differentiation medium for three weeks (lineage Z01B). (b) Analyzed in the cultivated DPSCs in the osteogenic differentiation

medium for three weeks (opposite phenotype; lineage Z08B). (c) Analyzed in the non-differentiated DPSCs (lineage Z06B).

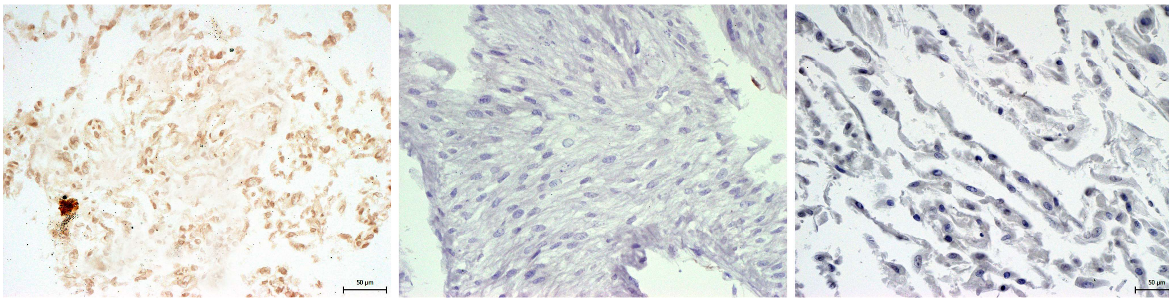

(**a**) (b) (c)

Figure S3: The osteocalcin (brown areas) in the produced extracellular mass. Scale bar 50 µm. (a) Analyzed in the cultivated DPSCs in the osteogenic differentiation medium for three weeks (lineage Z08B). (b) Analyzed in the cultivated DPSCs in the chondrogenic differentiation medium for three weeks (opposite phenotype; lineage Z01B). (c) Analyzed in the non-differentiated DPSCs (lineage Z06B).

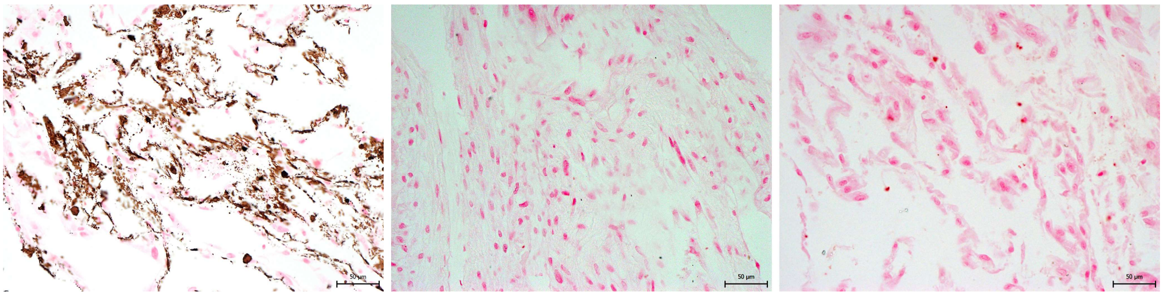

(**a**) (b) (c)

Figure S4: the calcium phosphate deposits (black or brown spots) in the extracellular mass. Scale bar 50 µm. (a) Analyzed in the cultivated DPSCs in the osteogenic differentiation medium for three weeks (lineage Z08B). (b) Analyzed in the cultivated DPSCs in the chondrogenic differentiation medium for three weeks (opposite phenotype, lineage Z01B), (c) Analyzed in the non-differentiated DPSCs (lineage Z06B).

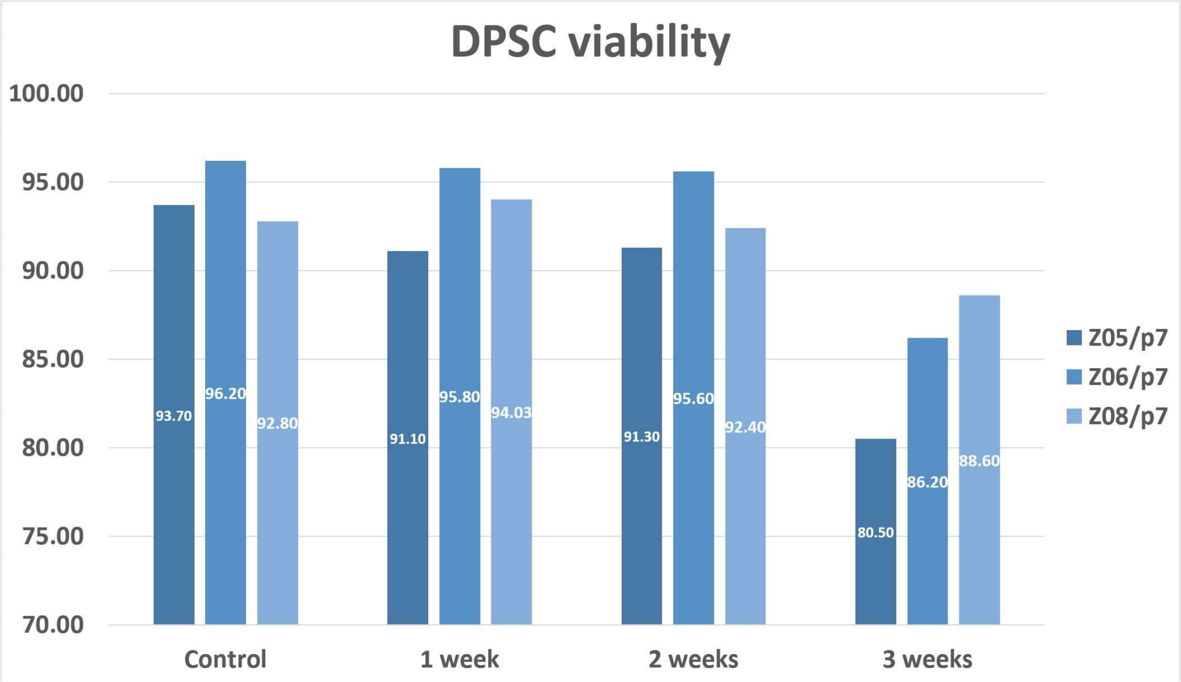

Figure S5: DPSC viability in % (lineages Z05/p7, Z06/p7, Z08/p7).
